# Psychiatric morbidity among resident doctors in a tertiary hospital in Nigeria

**DOI:** 10.4102/jcmsa.v2i1.91

**Published:** 2024-12-19

**Authors:** Mumeen O. Salihu, Alfred B. Makanjuola, Peter O. Ajiboye, Olatunji A. Abiodun

**Affiliations:** 1 Department of Behavioural Sciences, Kwara State University Teaching Hospital, Ilorin, Nigeria; 2 Department of Behavioural Sciences, University of Ilorin Teaching Hospital, Ilorin, Nigeria; 3 Department of Behavioural Sciences, Faculty of Clinical Sciences, University of Ilorin, Ilorin, Nigeria

**Keywords:** psychiatric morbidity, weighted prevalence, statistical weighting, resident doctors, tertiary hospital, UITH, Nigeria

## Abstract

**Background:**

Mental health concerns among trainee doctors are a pressing issue. Despite its importance, there is limited research on this topic, particularly in Nigeria. This study investigates the prevalence and correlates of psychiatric morbidity among resident doctors at the University of Ilorin Teaching Hospital.

**Methods:**

This two-phase cross-sectional descriptive study involved 176 trainee doctors across 16 medical specialities and subspecialities. The first phase involved screening for probable psychiatry ‘cases’ using the 12-item General Health Questionnaire (GHQ-12), and the second phase involved making specific psychiatric diagnoses using the Mini International Neuropsychiatric Interview (MINI plus). Statistical weighting was performed on the second phase data to estimate the weighted prevalence of psychiatric morbidity.

**Results:**

The mean age of respondents was 35.10 (s.d.: 4.07). The weighted prevalence of psychiatric morbidity among respondents was 35.2%, with generalised anxiety disorder (13.6%) being the most prevalent, while depression and opioid abuse accounted for 5.1% each. The presence of a stressful event within the previous 6 months (χ^2^ = 9.670; *p* = 0.002), poor sleep (χ^2^ = 6.822; *p* = 0.009), work-related stress (χ^2^ = 4.052; *p* = 0.044) and academic-related stress (χ^2^ = 11.735; *p* = 0.001) were significantly associated with psychiatric morbidity.

**Conclusion:**

There is a high prevalence of psychiatric morbidity in trainee doctors, with anxiety disorder being the most common mental health problem reported. Effective preventive strategies targeted at identified risk factors are encouraged to reduce its burden.

**Contribution:**

The poor mental health of resident doctors in this study highlights the urgent need to implement mental health-friendly policies at training institutions.

## Introduction

Mental health is a state of mental well-being that enables people to cope with the stresses of life, to realise their abilities, to learn well and work well, and to contribute to their communities.^1^ On the contrary, mental disorders are any health condition characterised by abnormal perception, thoughts, emotions, behaviour and relationships with others; this includes depression, bipolar disorder, schizophrenia and dementia, among others.^2^

Mental health issues, particularly depression and anxiety, have become pervasive workplace problems worldwide, resulting in significant consequences, including reduced productivity, absenteeism, high employee turnover and economic losses.^3^ A United Kingdom (UK) study estimated the total cost of mental health problems in staff to their employers at nearly £26 billion each year (equivalent to £1035 per employee in the UK workforce); £8.4bn from sickness absences; £15.1bn from reduced productivity at work and £2.4bn from staff turnover,^4^ while nearly $200bn was reported in 2018 as the total cost to employers in the United States by Greenberg et al.^5^ Promoting the mental well-being of employees can have some economic benefits in terms of staff retention, increased commitment and job satisfaction, improved productivity and performance, and reduced staff absenteeism.^6^

Compared to other professions, physicians have been identified as a group with a high risk of poor mental health, and trainee doctors are a particularly high-risk sub-group.^7^ Resident doctors are qualified physicians engaged in postgraduate medical training. The high-pressure environment of residency training can have profound psychological effects on resident doctors, including increased vulnerability to depression, burnout, anxiety disorders, substance abuse and suicidal ideation as they navigate the complexities of patient care, teaching and administration.^7,8,9^ However, like other physicians, trainee doctors are reluctant to disclose or seek psychiatric care from their primary care doctors. The lack of time, cost, confidentiality issues, stigma and licensure requirements, among others, were some of the common barriers reported by Aaronson et al.^10^ Other reported factors include denial of vulnerability (*omnipotent defence*) expressed by doctors and reinforced by the social structure of medical careers.^11^ This then raises a relevant question: If we cannot subdue our feeling of aversion to accept the truth of mental illness, what hope is there for the rest of society?^11^ There is, therefore, the need for regular mental health assessment of doctors and prompt treatment of mentally ill doctors in order to ensure improved quality care by doctors and the safety of the citizenry.

A systematic review of psychiatric morbidity among UK doctors between 1994 and 2012 found a prevalence rate ranging from 17% to 52% using the General Health Questionnaire (GHQ-12). The authors identified predictors such as neuroticism, low job satisfaction, high workload and long working hours.^12^ A survey of 8360 Australian doctors found a 28% prevalence rate of mental health issues based on GHQ-28.^13^ Factors linked to poor mental health included specialty type, medico-legal issues, lack of holiday in the previous year, long working hours, and neuroticism.^13^ Another study conducted on the mental health status of Saudi resident physicians showed a prevalence rate of 39.5% and 20.9% for anxiety and depression, respectively, using the Hospital Anxiety and Depression Scale (HADS). Factors such as younger age, being the leading family provider, poor sleep, exposure to injustice and long working hours were the predictors of psychiatric morbidity found in the study population.^14^

A study conducted among 629 early career doctors (resident doctors constitute the majority – 65.8%) working in 31 Nigerian public tertiary hospitals found that 30.6% had anxiety based on a 7-item General Anxiety Disorder (GAD-7) scale, with registrars being the most affected when compared to senior registrars.^15^ Adeolu et al.^16^ reported a 9.9% prevalence rate of psychiatric morbidity among junior doctors at the University College Hospital (UCH) in Ibadan, Nigeria, using the GHQ-12 (score > 2). The authors found that respondents who reported stress were approximately four times more likely to have poor mental health than those who did not. A prevalence rate of 14% was reported among trainee doctors at the University of Benin Teaching Hospital (UBTH), Nigeria, using the GHQ-28 (score ≥ 4).^17^ In comparison, a prevalence rate of 25.7% was found in resident doctors working at the University of Ilorin Teaching Hospital (UITH) Ilorin, Nigeria, based on GHQ-30 (score ≥ 4).^18^ Factors such as reduced religious activities, adverse effects on residents’ families, spousal dissatisfaction with the residency programme and increased stressful call duties were the identified predictors among resident doctors at UITH. Similarly, another study conducted in UITH 8 years later (in 2014) using the GHQ-12 (score of 3 or more) found a prevalence rate of 14.9%, and factors such as being married, non-participation in social activities and perceived heavy workload were found to be associated with psychiatric morbidity.^19^ Other stressors for poor mental health highlighted by previous studies include high patient workload, poor work environment, distant accommodation, a lack of recreational services within the hospital environment, long work hours, length of time spent in training, poor social support, complex patients and gender-related issues.^9,20^

Trainee doctors’ mental ill health can have profound effects on personal well-being, career prospects and relationships and may negatively affect patient outcomes.^21^ Specifically, evidence suggests that trainee doctors who suffered from depression were more prone to commit medical errors, with profound negative implications on patient safety and quality of care.^22^ It, therefore, means that the level of health and well-being of the resident doctors is a crucial indicator for the sustainability of healthcare systems, their performance and patient outcomes. Paradoxically, the Nigerian healthcare industry is witnessing mass emigration of doctors for greener pastures abroad, creating a critical deficit in human resources for health, particularly among junior doctors. This exodus has severe consequences, including increased workload and potential mental ill health for the remaining doctors.^23^ This necessitates the need for periodic mental health evaluation and addressing factors contributing to poor mental health among this group of doctors.

Furthermore, it is of note that previous local studies cited here used screening instruments such as GHQ and HADS to determine psychiatric morbidity. In this study, we aimed to determine the prevalence and specific psychiatric morbidities (like depression, anxiety, and substance use problems) and explore the associated demographic and work-related factors among trainee doctors working in different specialties at UITH, using a well-validated diagnostic tool (MINI-plus) after the initial screening with the GHQ-12. A nuanced understanding of these factors would assist in promoting mental well-being and developing sustainable preventive strategies to reduce associated burdens.

## Research methods and design

### Settings

The study was a section of a larger study conducted in the 16 departments offering residency training in UITH, Ilorin, Nigeria, between 01 October 2020 and 31 January 2021.^24^

### Study design

The study was a cross-sectional, observational survey conducted in two phases.

### Study population and sampling strategies

There were 245 total resident doctors at the time of the study and a total sampling method was employed. The inclusion criteria were residents who had spent at least 6 months in residency training at the time of the study and gave informed consent. The exclusion criteria included the respondents undergoing external rotation outside the hospital and those on treatment for established psychiatric illness. A total of 185 respondents were eligible and consented, but only 176 resident doctors participated by returning their questionnaires. Seven respondents failed to return their questionnaires, while two dropped out because of busy schedules. Each eligible respondent received a cover letter explaining the study’s purpose and provided written consent. They were recruited through their respective departments and grouped according to their training specialties for comparison. In each department, the resident doctors were listed and allocated numerical codes similar to that on the questionnaires.

### Instruments

These included a designed questionnaire on socio-demographic and work-related factors, GHQ-12 and MINI-plus (MINI-plus 5.0.0, English version).

The GHQ-12 was developed by David Goldberg as a self-administered screening instrument for detecting psychological morbidity in primary care, general medical practice or community surveys. However, it does not specify the exact nature of mental disorders.^25^ In some studies, the GHQ-12 has a sensitivity of 83.7% and specificity of 79%, with a modal optimum score of 2 – 3.^26^ It has been reported to have inter-rater reliability and internal consistency with Cronbach’s alpha of 0.76.^27^ The GHQ-12 had been used locally, where the conventional cut-off points of ≥ 3 were used to categorise probable psychiatric morbidity.^26^ However, in this study, the GHQ-12 score of ≥ 3 was used as probable cases (GHQ-positive), while a score of ˂ 3 was taken as probable non-cases (GHQ-negative).^26^ The questionnaire takes about 2 min – 3 min to complete and has two scoring systems – bi-modal scoring (0-0-1-1) and Likert scoring (0-1-2-3). The bi-modal scoring system was used in this study.

The MINI International Neuropsychiatric Interview is a brief diagnostic interview based on the Diagnostic and Statistical Manual, fourth edition (DSM–1V), and the International Classification of Diseases, tenth edition (ICD-10), which includes the main Axis 1 disorders.^28^ This instrument assesses mental disorders throughout an individual’s life, and the diagnostic categories’ reliability was 0.86 to 1 with an application time of 15 min – 30 min.^28^ The instrument has been used and validated in Nigeria.^29^ The MINI-plus was used to assess specific psychiatric disorders, which include depressive disorders, anxiety disorders and substance use problems among respondents in this study. The diagnosis was based on ICD-10 diagnostic criteria.

### Procedure

The assessment was in two stages.

*Stage one*: After reading the information sheet and signing the consent form, each respondent was given a designed questionnaire and GHQ-12 by the research assistant to complete.

*Stage two*: This involved the use of MINI-plus by the researcher to determine the prevalence of psychiatric morbidity and specific psychiatric diagnosis based on ICD-10 diagnostic criteria. The research assistant randomly selected 10% of respondents who were GHQ-negative (i.e., probable non-cases) and all (100%) those who were GHQ-positive (i.e., probable cases) from the first stage. In selecting 10% of the GHQ-negative respondents, the first GHQ-negative subject was selected using a simple random technique, and subsequent GHQ-negative respondents were selected using a systematic random sampling method until the quota was satisfied. All (100%) probable cases and 10% probable non-cases were then interviewed using MINI-plus by the researcher to determine specific psychiatric disorders. The researcher was thus blind to the first-stage GHQ-12 scores of the respondents.

### Data analysis

All data collected were analysed using the Statistical Package for Social Sciences version 27 (SPSS 27). Mean ± standard deviation (s.d.) was used for continuous variables, while categorical variables were interpreted as proportions and percentages. Chi-square was used to compare associations between psychiatric morbidity and the independent variables, and significance was set as a *p* ˂ 0.05.

The weighted prevalence of psychiatric morbidity was determined using the statistical weighting method, as recommended for two-phase epidemiological surveys in psychiatric research.^30^ The weighted prevalence rates aim to extrapolate mathematically to the total study population what the prevalence figures (total psychiatric morbidity and prevalence rates for the different psychiatric disorders) would have been by taking the weighted sum of the prevalence of ‘true’ cases within each of the strata defined by the first-phase screening questionnaire. The stratum weight reflects its relative size (i.e., first-phase stratum weights sum to 1). The overall prevalence estimate is the sum of the products of the stratum prevalence and stratum weights over all first-phase strata, summarised using the algebraic notation (Horvitz-Thompson estimator)^30^:

Π – Σwiyi/Σwi

Π – denoting estimate of the prevalence

Σ – ‘the sum of’

wi – the subject’s sampling weight

yi –the second-stage subject: yi is one if it is a ‘true’ case or 0 if otherwise

*wiyi* – is the estimated number of first-phase ‘true’ cases (i.e. the sum of the product of the second-phase ‘true’ case and the sampling weight).

### Ethical considerations

The UITH Ethics and Research Committee (ERC) approved the study with reference number ERC PAN/2020/06/0023. Respondents were fully informed about the study’s objectives and benefits and provided informed consent. Confidentiality was ensured and respondents identified with psychiatric issues received counselling and referral for further support

## Results

### Characteristics of the respondents

Of the 185 respondents who were eligible and participated in the survey, only 176 (95.1%) responded. The mean age of respondents was 35.10 ± 4.07 years; 53 (30.1%) were females, 79 (44.9%) were registrars, and only 26 (14.8%) were single. Sixty (34.1%) and 155 (88.1%) respondents were satisfied with their income and relationship with their colleagues, respectively. Twenty-two (12.5%) residents were receiving treatment for one or more chronic medical illnesses, with systemic hypertension ranking the highest (31.8%). The average weekly mean duration of work hours was 55.21 ± 28.06. Respondents with poor sleep accounted for 52.8%, with an average of 2.92 ± 1.53 days of restorative sleep per week. According to this study, sleeping well means good sleep (perceived restorative sleep for at least 4 days a week), while poor sleep (not sleeping well) is perceived restorative sleep for fewer than 4 days a week.^24^ Most (82.4%) respondents experienced major stressful events within the preceding 6 months, and 96 (54%) had work-related stress (see Table 1 and Table 2).

**TABLE 1:** Socio-demographic, residency and work-related characteristics of respondents (*N* = 176).

Variables	Frequency (*n*)	Percentage	Mean ± s.d.	Range
**Age groups (years)**	-	-	35.10 ± 4.07	26–48
≤ 30	23	13.1	-	-
31–35	72	40.9	-	-
36–40	70	39.8	-	-
≥ 41	11	6.2	-	-
**Gender**				
Female	53	30.1	-	-
Male	123	69.9	-	-
**Marital status**				
Single	26	14.8	-	-
Married living together	123	69.9	-	-
Married living apart	27	15.3	-	-
**Number of children (*n* = 155)**				
None	20	12.9	-	-
1–2	84	54.2	-	-
> 2	51	32.9	-	-
**Monthly income ₦ (in thousands) (*n* = 176)**				
< 250	11	6.3	-	-
250 to < 350	88	50.0	-	-
350 to 450	55	31.3	-	-
> 450	22	12.5	-	-
**Satisfaction with income**				
No	116	65.9	-	-
Yes	60	34.1	-	-
**Rank**				
Registrar	79	44.9	-	-
Senior registrar	97	55.1	-	-
**Take calls**				
Yes	174	98.9	-	-
No	2	1.1	-	-
**Duration of work per week (hours)**	-	-	55.21 ± 28.06	-
≤ 50	99	56.3	-	-
> 50	77	43.7	-	-
**Break during working hours**				
Yes	34	19.3	-	-
No	142	80.7	-	-
**Sleeps well**				
Yes	83	47.2	-	-
No	93	52.8	-	-
**Duration of training (years)**				
One	20	11.4	-	-
Two	54	30.7	-	-
Three	24	13.6	-	-
Four	20	11.4	-	-
Five	27	15.3	-	-
Six or more	31	17.6	-	-

s.d, standard deviation; ₦, Nigerian naira.

**TABLE 2:** Stressors reported by resident doctors (*N* = 176).

Variables	Yes		No	
	*n*	%	*n*	%
Stressful job environment	133	75.6	43	24.4
Accommodation away from hospital is stressful	92	52.3	84	47.7
Comfortable call room	58	33.0	118	67.0
Share consulting room	121	8.8	55	31.2
Presence of major stressful events within the previous 6-month	145	82.4	31	17.6
Work-related stress	96	54.5	80	45.5
Family-related stress	27	15.3	149	84.7
Financial-related stress	38	21.6	138	78.4
Academics-related stress	52	29.5	124	70.5
Attempted exams	105	59.7	71	40.3
Satisfaction with relationship with colleagues	155	88.1	21	11.9
Satisfaction with training programme	36	20.5	140	79.5

### Prevalence of psychological distress among respondents using 12-item General Health Questionnaire

This study shows that 51 (29%) resident doctors had psychological distress on GHQ-12 (i.e., GHQ-12 positive).

### Prevalence and pattern of psychiatric morbidity among respondents based on MINI interview

This study shows that 36 (20.5%) respondents had psychiatric morbidity after a MINI interview (unweighted psychiatric morbidity).

Fifteen (8.5%) respondents had GAD, 9(5.1%) experienced depression, 4 (2.3%) had both depression and GAD, 1 (0.6%) each had panic disorder and opioid abuse, while 6 (3.4%) were categorised as ‘others’ (see Table 3).

**TABLE 3:** Weighted and unweighted psychiatric morbidity rates in the study population.

Variables	Before weighting		After weighting	
	*n*	%	*n*	%
Overall prevalence	36	20.5	62	35.2
**Types of psychiatric disorders**				
GAD	15	8.5	24	13.6
Depression	9	5.1	9	5.1
GAD + depression	4	2.3	4	2.3
Panic disorder	1	0.6	10	5.7
Opioid abuse	1	0.6	9	5.1
Others†	6	3.4	6	3.4

GAD, generalised anxiety disorder.

† , Include two cases of generalised anxiety disorder (GAD) and depression with comorbid caffeine dependence and one case each of social anxiety disorder, alcohol abuse, caffeine dependence and depression with comorbid alcohol abuse.

### Estimated prevalence and pattern of psychiatric morbidity among respondents after weighting

Table 3 shows that 62 (35.2%) respondents had psychiatric morbidity, while 114 (64.8%) had no psychiatric morbidity following weighting.

After weighting, 24 (13.6%) respondents had GAD, 10 (5.7%) had panic disorder, 9 (5.1%) experienced depression, 9 (5.1%) had opioid abuse, 4 (2.3%) had both depression and GAD, and 6 (3.4%) constituted ‘others’.

### Associations between socio-demographic variables and psychiatric morbidity

There was no significant association between socio-demographic variables and the presence of psychiatric morbidity. Although more female resident doctors had psychiatric morbidity than male resident doctors (26.4% vs. 17.9%), the difference was not statistically significant. Also, respondents who were satisfied with their monthly income had less psychiatric morbidity than those dissatisfied with their income (18.3% vs. 21.6%; χ^2^ = 0.252, *p* = 0.616), but this did not reach the level of statistical significance (Table 4).

**TABLE 4:** Association between socio-demographic variables and psychiatric morbidity.

Variables	Psychiatric morbidity				χ^2^	*p*
	MINI-positive (*n* = 36)		MINI-negative (*n* = 140)			
	*n*	%	*n*	%		
**Age groups (years)**	-	-	-	-	6.447	0.092
≤ 30	9	39.1	14	60.9	-	-
31 – 35	12	16.7	60	83.3	-	-
36 – 40	14	20.0	56	80.0	-	-
≥ 41	1	9.1	10	90.9	-	-
**Gender**	-	-	-	-	1.656	0.198
Female	14	26.4	39	73.6	-	-
Male	22	17.9	101	82.1	-	-
**Marital status**	-	-	-	-	1.654	0.437
Single	5	19.2	21	80.8	-	-
Married living together	23	18.7	100	81.3	-	-
Married living apart	8	29.6	19	70.4	-	-
**Monthly income (₦) (in thousands)**	-	-	-	-	1.020	0.796
< 250	3	27.3	8	72.7	-	-
250 to < 350	19	21.6	69	78.4	-	-
350 to 450	9	16.4	46	83.6	-	-
> 450	5	22.7	17	77.3	-	-
**Satisfaction with income**	-	-	-		0.252	0.616
No	25	21.6	91	78.4	-	-
Yes	11	18.3	49	81.7	-	-
**Departments**	-	-	-	-	1.838	0.399
Laboratories	3	30.0	7	70.0	-	-
Medicals	19	23.5	62	76.5	-	-
Surgical	14	16.5	71	83.5	-	-
**Duration of training (years)**	-	-	-	-	4.753	0.447
One	7	35.0	13	65.0	-	-
Two	11	20.4	43	79.6	-	-
Three	3	12.5	21	87.5	-	-
Four	3	15.0	17	85.0	-	-
Five	7	25.9	20	74.1	-	-
Six or more	5	16.1	26	83.9	-	-

χ^2^, Chi-square test; ₦, Nigerian naira.

Note: *p* < 0.05 is statistically significant.

### Associations between work-related factors and psychiatric morbidity

There was a significant association between psychiatric morbidity and resident doctors who reported having poor sleep (χ^2^ = 6.822; *p* = 0.009), those reporting stressful events within the previous 6 months (χ^2^ = 9.670; *p* = 0.002), those with work-related stress (χ^2^ = 4.054; *p* = 0.044) and academic-related stress (χ^2^ = 11.735; *p* = 0.001). It was observed that 24.1% of registrars versus 17.5% of senior registrars had psychiatric morbidity, although the difference was not statistically significant. In addition, the duration of weekly work hours, including call hours, access to breaks during working hours, attempts at examination, work-related stress and reporting of medical errors were not significantly associated with psychiatric morbidity (Table 5).

**TABLE 5:** Relationships between work-related factors and psychiatric morbidity.

Variables	Psychiatric morbidity				χ^2^	*p*
	MINI-positive (*n* = 36)		MINI-negative (*n* = 140)			
	*n*	%	*n*	%		
**Rank**	-	-	-	-	1.139	0.286
Registrar	19	24.1	60	75.9	-	-
Senior registrar	17	17.5	80	82.5	-	-
**Sleep well**	-	-	-	-	6.822	0.009
Yes	10	12.0	73	88.0	-	-
No	26	28.0	67	72.0	-	-
**Presence of stressful events within the last 6 months**	-	-	-	-	9.670	0.002
Yes	36	24.8	109	75.2	-	-
No	0	0.0	31	100.0	-	-
**Satisfaction with relation with colleague**	-	-	-	-	0.966	0.326
Yes	30	19.4	125	80.6	-	-
No	6	28.6	15	71.4	-	-
**Satisfaction with training programme**	-	-	-	-	2.428	0.119
Yes	4	11.1	32	88.9	-	-
No	32	22.9	108	77.1	-	-
**Duration of work per week (hours)**	-	-	-	-	0.718	0.397
≤ 50	18	18.2	81	81.8	-	-
> 50	18	23.4	59	76.6	-	-
**Work-related stress**	-	-	-	-	4.052	0.044
Yes	25	26.0	71	74.0	-	-
No	11	13.8	69	86.2	-	-
**Academic-related stress**	-	-	-	-	11.735	0.001
Yes	19	36.5	33	63.5	-	-
No	17	13.7	107	86.3	-	-
**Attempts at exams**	-	-	-	-	2.381	0.123
Yes	17	16.5	86	83.5	-	-
No	19	26.0	54	74.0	-	-
**Medical errors**	-	-	-	-	5.556	0.062
Sometimes	15	31.3	33	68.8	-	-
Rarely	20	17.5	94	82.5	-	-
Never	1	7.1	13	92.9	-	-

χ*^2^*, Chi-square test.

Note: *p* < 0.05 is statistically significant.

## Discussion

### Prevalence of psychiatric morbidity among resident doctors

Poor mental health among doctors is a severe cause for concern, particularly among trainee doctors, because of its wide-range implications.^22^ The prevalence of psychiatric morbidity in this study was 35.2% and this is similar and compares to the prevalence of 34% and 37.1% reported among trainee doctors and emergency physicians in the UK and China, respectively.^31,32^

The prevalence of 35.2% is higher than those reported in Nigerian studies among resident doctors in Benin and Ilorin, where 14% and 25.7% rates were revealed, respectively.^17,18^ This may be because of the differences in methodology, as the two studies only used a screening tool (GHQ) compared to our study, which went a step further to determine the prevalence rate using the diagnostic tool – *MINI plus*. The stressful work environment, as reported by most respondents, might have contributed to the high rate of mental health problems seen in our study. Furthermore, this study coincided with the second wave of the coronavirus disease 2019 (COVID-19) pandemic, and this could have contributed to the rates of mental disorders observed in the study. However, this study did not examine specific questions about the COVID-19 pandemic.

### Factors associated with psychiatric morbidity among resident doctors

#### Socio-demographic factors

There is a paucity of evidence on the effects of socio-demographic factors such as age, gender and marital status on the mental health of resident doctors.^12^ While some studies reported that being a female and married were predictive of psychiatric morbidity,^19,33,34^ this study, however, found no significant associations between the presence of psychiatric morbidity and socio-demographic variables. Although resident doctors (irrespective of gender) had a uniform work structure, one study reported that female doctors in residency training were more likely to disclose psychological problems and seek help than male doctors.^35^

#### Work-related factors

This study found that the presence of poor sleep, work-related stress, academic-related stress and a positive history of major stressful events within the previous 6 months was significantly associated with psychiatric morbidity. Specifically, psychiatric morbidity was twice as common among respondents who reported poor sleep compared to those who did not in this study. Refreshing sleep is crucial as it relates directly to physical and mental wellness. Jaradat et al.^36^ found a strong relationship between poor sleep quality and psychiatric disorders such as depression and anxiety among resident physicians. Similarly, Pokhrel *et al*.^33^ reported that adequate sleep was a significant protective correlate for anxiety among resident doctors. Although the protective effect of sleep was present, the study included resident doctors and medical students compared to only resident doctors in our study.

Also, the presence of work-associated stress and academic-related stress was significantly associated with psychiatric morbidity in this study. However, this is unsurprising as residency training involves clinical and academic work. Residency training exposes young physicians to harsh environmental conditions and stress, which include sleep deprivation, heavy workload, long work hours, uncontrolled schedules, inadequate personal time, complex patients, deprived learning environment, isolation and other social problems.^9,37^ As a result, resident doctors are prone to developing poor mental health, like depression, anger control problems and substance abuse.^9,38^ Some authors have found a strong association between perceived high job stress and psychiatric morbidity among doctors.^19,39,40^ Tyssen and colleagues in Norway found that recent major life events, such as partner relationship problems and the death of family members or close friends, among others, were predictive of psychiatric morbidity.^40^ This is consistent with the findings in our study, where all respondents who experienced any major stressful events within the last 6 months had a significant association with psychiatric morbidity. There is, therefore, a need for the implementation of stress-preventive strategies and the promotion of a sleep hygiene culture through appropriate modification of the clinical work process to protect trainee doctors’ mental health for improved productivity, quality care and citizen safety.

### Strength and limitations

Our study helps to expand and update previous local studies^18,19^ on this important topic. We used standardised, well-validated diagnostic tools to determine the psychiatric morbidity, including specific psychiatric disorders. Likewise, the response rate of 95.1% was high, and the findings are expected to be a factual representation of the study population. A standard weighting method was used to compute the estimated prevalence of psychiatric morbidity.

The limitation of this study is its cross-sectional design, where the temporal relationship between demographic and work-related stressors and psychiatric morbidity becomes difficult to establish. Also, there is the possibility of response bias as some participants may respond in socially desirable ways to self-administered questionnaires and MINI interviews, which could lead to underreporting because of concern for stigma. However, this was minimised by ensuring confidentiality issues, and respect for privacy was adhered to during the interview in the second phase of the study. The recall bias in the self-reported questionnaires used in this study was also a possible limitation. Longitudinal studies can be explored to confirm our findings.

## Conclusion and recommendations

This study reported a high prevalence of psychiatric morbidity among resident doctors comparable to other previous studies, with anxiety disorder being the most common mental health problem reported. The identifiable determinants were the presence of poor sleep, work-related stress, academic-related stress and a positive history of major stressful events within the previous 6 months. The resident doctors need to be aware of their vulnerability to poor mental health and the attendant negative impact on the care (poor quality of care) of their patients. Therefore, there is a need for periodic mental health screening and prompt targeted interventions where necessary. Stress management training at regular intervals should be institutionalised to reduce stress in the workplace, and relevant stakeholders should try to create a more conducive work environment for doctors. A psychological support helpline available 24/7 (operated by selected mental health experts) should be created for resident doctors and, by extension, other staff, as this may improve access and the mental health-seeking behaviour of doctors. Future studies should involve a large-scale multi-centre longitudinal research work across six geopolitical zones in Nigeria to come up with generalisable findings for proper national planning and implementation of mental health-friendly policies at the workplace, anchored on better welfare, suitable working environments and quality training that will guarantee better outcome and optimum satisfaction.
